# Genes That Predict Poor Prognosis in Breast Cancer via Bioinformatical Analysis

**DOI:** 10.1155/2021/6649660

**Published:** 2021-04-17

**Authors:** Qian Zhou, Xiaofeng Liu, Mingming Lv, Erhu Sun, Xun Lu, Cheng Lu

**Affiliations:** ^1^Department of Breast, Women's Hospital of Nanjing Medical University, Nanjing Maternity and Child Health Care Hospital, Nanjing 210004, China; ^2^School of Public Health, Yale University, New Haven, CT 06520, USA

## Abstract

**Background:**

Breast cancer is one of the most commonly diagnosed cancers all over the world, and it is now the leading cause of cancer death among females. The aim of this study was to find DEGs (differentially expressed genes) which can predict poor prognosis in breast cancer and be effective targets for breast cancer patients via bioinformatical analysis.

**Methods:**

GSE86374, GSE5364, and GSE70947 were chosen from the GEO database. DEGs between breast cancer tissues and normal breast tissues were picked out by GEO2R and Venn diagram software. Then, DAVID (Database for Annotation, Visualization, and Integrated Discovery) was used to analyze these DEGs in gene ontology (GO) including molecular function (MF), cellular component (CC), and biological process (BP) and Kyoto Encyclopedia of Gene and Genome (KEGG) pathway. Next, STRING (Search Tool for the Retrieval of Interacting Genes) was used to investigate potential protein-protein interaction (PPI) relationships among DEGs and these DEGs were analyzed by Molecular Complex Detection (MCODE) in Cytoscape. After that, UALCAN, GEPIA (gene expression profiling interactive analysis), and KM (Kaplan–Meier plotter) were used for the prognostic information and core genes were qualified.

**Results:**

There were 96 upregulated genes and 98 downregulated genes in this study. 55 upregulated genes were selected as hub genes in the PPI network. For validation in UALCAN, GEPIA, and KM, 5 core genes (*KIF4A*, *RACGAP1*, *CKS2*, *SHCBP1*, and *HMMR*) were found to highly expressed in breast cancer tissues with poor prognosis. They differentially expressed between different subclasses of breast cancer.

**Conclusion:**

These five genes (*KIF4A*, *RACGAP1*, *CKS2*, *SHCBP1*, and *HMMR*) could be potential targets for therapy in breast cancer and prediction of prognosis on the basis of bioinformatical analysis.

## 1. Introduction

Breast cancer is one of the most commonly diagnosed cancers all over the world, and it is now the leading cause of cancer death among females; incidence rates for breast cancer far exceed those for other cancers in both transitioned and transitioning countries [1]. The causes of breast cancer are related to both hereditary and genetic factors such as gender, age, family history, and hormone therapy. One of the major hallmarks of cancer is the disorder of gene expression [2, 3]. RNA is a critical factor for gene expression in the development of cancer. It has various forms including protein-coding mRNA and noncoding RNAs, for example, lncRNAs and miRNAs. Recent studies show that processing of RNA is changed in cancer [4]. Different genetic conditions such as the different gene expressions (DGEs) can lead to different individualized treatment and different effects of treatment. Bioinformatical analysis is a method using gene chips in public database to analyze the characters of one type of disease; it can help researchers in better understanding the molecular mechanism behind different types of cancer [5–7], find new potential targets of early diagnosis and therapy [8, 9], or discover new biomarkers for prognostic predictor [10, 11]. At present, there are several predictive biomarkers for breast cancer, for example, triple-negative breast cancers (TNBC) which lack estrogen receptor (ER-), progesterone receptor (PR-), and amplification of human epidermal growth factor receptor 2 (HER2-). TNBC often has a poor therapeutic response and a poor prognosis [12]; we must find more biomarkers to help us predict the prognosis of TNBC and targets to cure the disease. The rapid development of biological and biomedical research made all this came true. In our study, with the help of bioinformatical analysis, we extended the knowledge related to breast cancer based on various large databases for conducting genes that predict poor prognosis in breast cancer especially TNBC.

## 2. Materials and Methods

### 2.1. Microarray Data Information

In our study, the gene expression profiles were downloaded from the GEO database (Gene Expression Omnibus) which was a free public database that contained many genes or microarray profiles (https://www.ncbi.nlm.nih. gov/geo/). We chose GSE86374, GSE5364, and GSE70947 for use and got their expression of breast cancer and normal breast tissues [13]. GSE86374 was based on GPL6244 platform ([HuGene-1_0-st] Affymetrix Human Gene 1.0 ST Array [transcript (gene) version]), GSE5364 was based on GPL96 platform ([HG-U133A] Affymetrix Human Genome U133A Array), and GSE70947 was based on GPL13607 platform (Agilent-028004 SurePrint G3 Human GE 8x60K Microarray).

### 2.2. Data Preprocessing and Analyzing of DEGs

All raw data were processed by the online tool GEO2R (https://www.ncbi.nlm. http://nih.gov/geo/geo2r/) from GEO. Genes which met the cutoff criteria with adjusted *P* value > 0.05 and ∣logFC | ≥1.0 were considered as DEGs. If the DEGs were with logFC > 0, we considered them as upregulated genes; on the contrary, if the DEGs were with logFC < 0, we considered them as downregulated genes. After screening, we changed GB-ACC in GSE70947 into gene symbol. Subsequently, data were checked in Venn software online to look for common DEGs among GSE86374, GSE5364, and GSE70947 (http://bioinformatics.psb.ugent.be/webtools/Venn/).

### 2.3. Analysis of DEGs in GO Enrichment and KEGG Pathway of Breast Cancer

GO analysis which means gene ontology analysis now is prevalently used to define genes or RNA, and it is a very useful method for our daily large scale of functional enrichment research. GO analysis can be classified into different gene functions, for example, biological process (BP), molecular function (MF), and cellular component (CC). KEGG stores a lot of data about biological pathways, diseases, and chemical substances and is widely used nowadays. In our study, we used the database for DAVID (https://david.ncifcrf.gov/) to analyze DEG enrichment of BP, CC, and MF and the KEGG pathways. *P* < 0.01 was considered statistically significant.

### 2.4. Protein-Protein Interaction (PPI) Network and Node Analysis in Breast Cancer

STRING is an online tool whose full name is Search Tool for the Retrieval of Interacting Genes (https://string-db.org/) [14]. We used it to get information of interaction between proteins (*medium confident 0.4*). Then, we used the app Cytoscape to examine correlation among them and find central genes in the PPI network (degree cutoff = 2, node score cutoff = 0.2, K-core = 2, max. depth = 100).

### 2.5. Survival Analysis of Core Genes in Breast Cancer

UALCAN is a comprehensive web resource for analyzing cancer data [15] (http://ualcan.path.uab.edu/index.html), and GEPIA is an online tool for gene expression profiling interactive analysis as well (http://gepia.cancer-pku.cn/). They can both provide graphs and plots depicting gene expression and patient survival information based on gene expression. KM is an online survival analysis tool to rapidly assess the effect of certain genes on cancer prognosis using microarray data (https://kmplot.com/analysis/index.php?p=service&cancer=breast) [16]. In our study, we first searched all the hub genes on the UALCAN website to identify the ones with poor survival. Then, we used GEPIA to rerecognize whether these core genes have different expressions between breast cancer and normal breast tissues. After that, we used the KM plotter to identify their OS and RFS among breast cancer patients.

### 2.6. Reanalysis of Genes on UALCAN

We reanalyzed core genes based on different subclasses of breast cancer and their correlation using UALCAN.

## 3. Results

### 3.1. Microarray Data Information

Three profiles (GSE86374, GSE5364, and GSE70947) were chosen from the GEO database in our study. GSE86374 included 124 breast cancer samples and 35 normal breast samples, GSE5364 included 183 breast cancer samples and 13 normal breast samples, and GSE70947 included 148 breast cancer samples and 148 normal breast samples. There were totally 455 breast cancer samples and 196 normal breast samples in our study (Table 1).

### 3.2. Identification of DEGs in Breast Cancer

**Figure 1:**
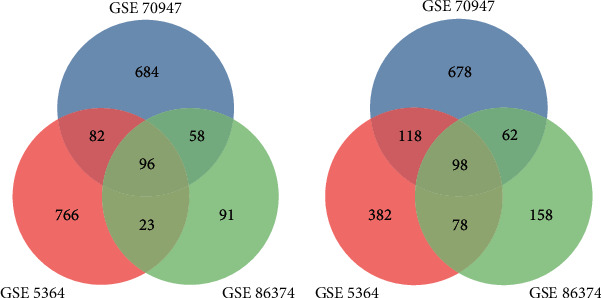
Venn diagram of DEGs common to all three GEO datasets: (a) upregulated genes; (b) downregulated genes.

We used GEO2R online tools to get DEGs in three datasets. Based on the criteria of adjusted *P* value > 0.05 and ∣logFC | ≥1.0, there were 268 upregulated and 396 downregulated genes in GSE86374, 967 upregulated and 676 downregulated genes in GSE5364, and 920 upregulated and 956 downregulated ones in GSE70947. Subsequently, Venn diagram software online was performed to identify common DEGs in these three different datasets. We found 194 DEGs expressed significantly differentially among all three groups, 96 significantly upregulated and 98 downregulated (Figure 1).

### 3.3. Analysis of GO Enrichment and KEGG Pathway in Breast Cancer

**Figure 2:**
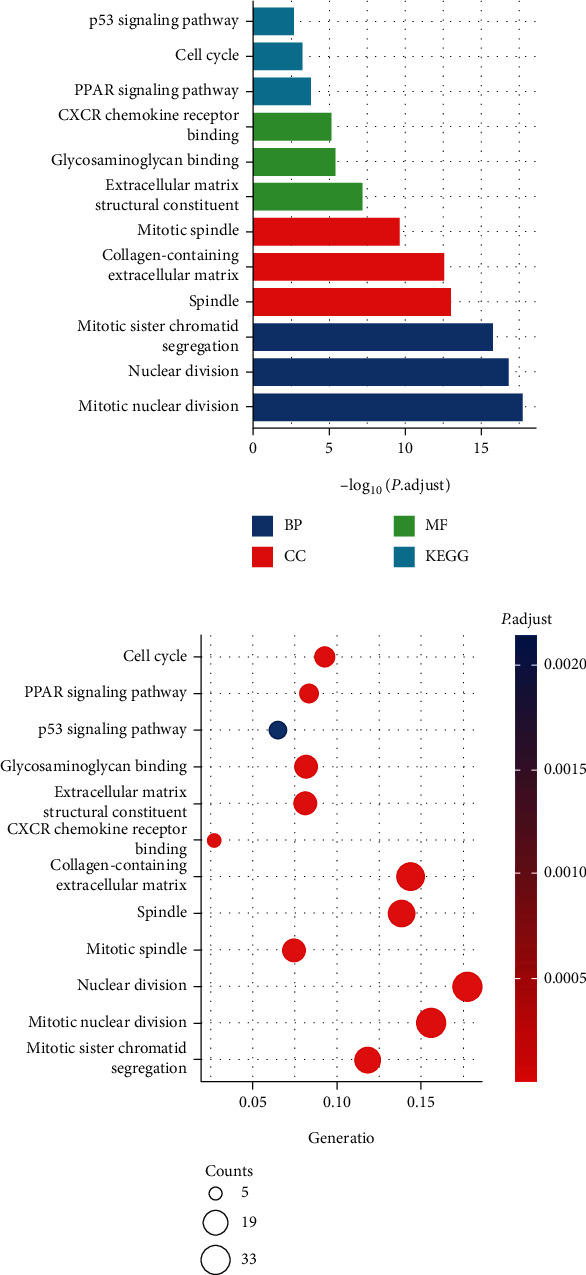
GO function enrichment and KEGG pathway analysis of DEGs (*P* < 0.01, count > 5, DAVID).

We analyzed all the 194 DEGs using DAVID for GO enrichment analysis, and results showed the following. (1) In biological processes (BP), DEGs were mainly enriched in microtubule-based movement, cell adhesion, collagen fibril organization, cerebral cortex development, chemokine-mediated signaling pathway, cellular response to amino acid stimulus, cellular response to lipopolysaccharide, activation of protein kinase activity, negative regulation of smooth muscle cell proliferation, mitotic cytokinesis, positive regulation of cytokinesis, mitotic spindle assembly, regulation of attachment of spindle microtubules to kinetochore, positive regulation of cholesterol storage, positive regulation of macrophage-derived foam cell differentiation, lipoprotein transport, mitotic spindle assembly checkpoint, and collagen catabolic process. (2) In cell component (CC), DEGs were mainly enriched in extracellular exosome, extracellular space, proteinaceous extracellular matrix, perinuclear region of cytoplasm, midbody, kinesin complex, extracellular matrix, sarcolemma, kinetochore, mitotic spindle, spindle microtubule, and centralspindlin complex. (3) In molecular function (MF), DEGs were mainly enriched in ATP binding, calcium ion binding, heparin binding, metalloendopeptidase activity, ATPase activity, microtubule motor activity, ATP-dependent microtubule motor activity, plus-end-directed, drug binding (*P* < 0.01). Then, we analyzed these DEGs in the KEGG pathway. Results showed that DEGs were mainly in the PPAR signaling pathway, cell cycle, ECM-receptor interaction, p53 signaling pathway, oocyte meiosis, pathways in cancer, focal adhesion, cytokine-cytokine receptor interaction, and progesterone-mediated oocyte maturation (Figure 2, *P* < 0.01).

### 3.4. Protein-Protein Interaction (PPI) Network and Node Analysis in Breast Cancer

**Figure 3:**
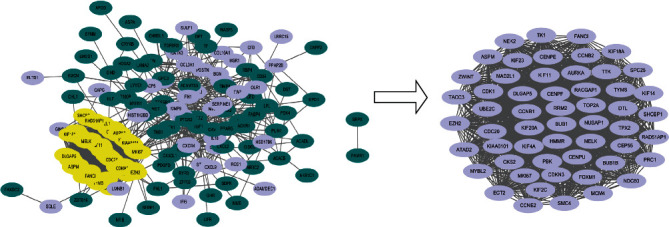
DEG protein-to-protein interaction network constructed by STRING and Cytoscape analysis. (a) There were 192 nodes and 2025 edges in the PPI network. Nodes meant proteins and edges meant the interaction of the proteins. Purple circles meant upregulated genes and green circles meant downregulated ones; yellow circles were highlighted hub genes. (b) Hub genes of DEGs (degree cutoff = 2, node score cutoff = 0.2, K-core-2, max depth = 100).

We imported these total DEGs into STRING and got information of interaction between proteins. There were 192 nodes and 2025 edges in the PPI network. We used Cytotype for further study. With the help of MCODE in Cytotype, we got 55 hub genes among these nodes, all of which were upregulated genes (Figure 3, *P* < 0.05).

### 3.5. Analysis of Central Genes by UALCAN, GEPIA, and KM Plotter

**Figure 4:**
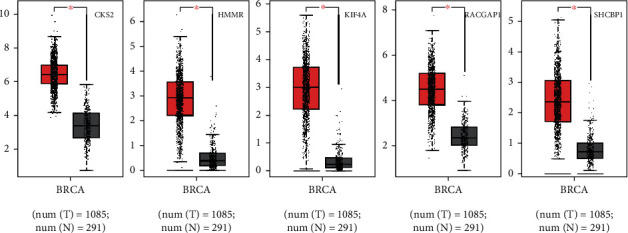
Five core genes in breast cancer patients compared to healthy people. GEPIA was used to further identify the expression level of these genes between breast cancer and normal people. All the 5 genes had significant high levels in breast cancer specimen. Red means tumor and grey means normal tissues (*P* < 0.05).

**Figure 5:**
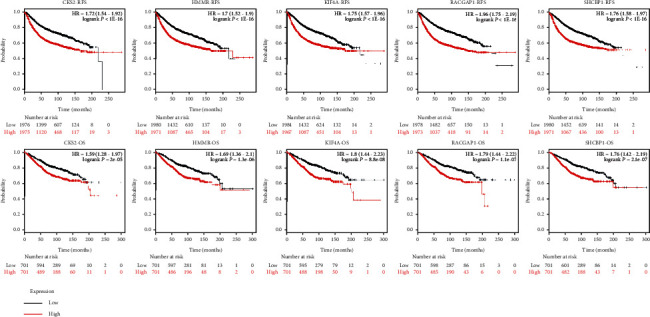
Prognostic information of these genes. KM plotter was used to identify the prognostic information of core genes, and all of them had a significantly worse survival. OS: overall survival; RFS: relapse-free survival; *P* < 0.05.

UALCAN analyzed all the 55 genes; results showed that 5 of them were with a significantly worse survival (Table 2, *P* < 0.01). They are *CKS2*, *HMMR*, *KIF4A*, *RACGAP1*, and *SHCBP1*. Then, we used GEPIA to dig up their expression level between breast cancer and normal breast tissues. Results revealed all these genes with high expression in breast cancer. Then, we reanalyzed these core genes on KM plotter; results showed that they all had a significant poor survival (Figures 4 and 5, *P* < 0.05).

### 3.6. Reanalysis of Genes on UALCAN

**Figure 6:**
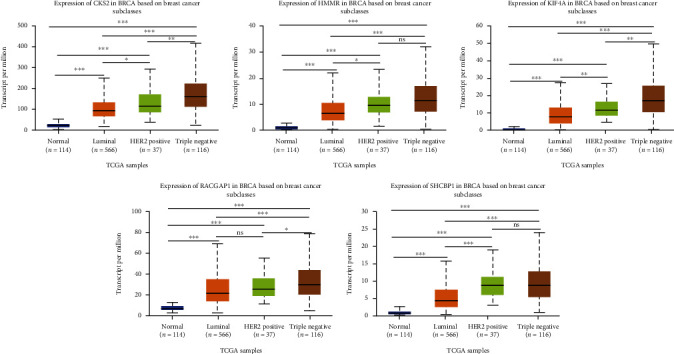
Expression of core genes in breast cancer on different subclasses (UALCAN).

**Figure 7:**
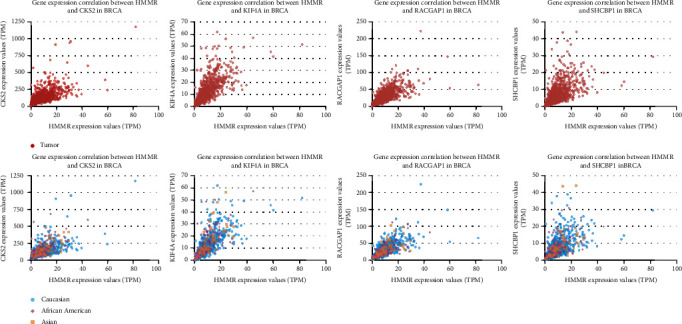
*CKS2*, *KIF4A*, *RACGAP1*, and *SHCBP1* all have positive correlation with *HMMR* (UALCAN).

We reanalyzed the core genes in different subclasses of breast cancer in UALCAN. Results showed that these five genes had different expressions among breast cancer subclasses including TNBC (Figure 6). *CKS2*, *KIF4A*, *RACGAP1*, and *SHCBP1* all have positive correlation with *HMMR* (Figure 7). The correlation between *HMMR* and the other four genes is 0.62, 0.73, 0.71, and 0.59, respectively.

## 4. Discussion

In our research, we studied GSE5364, GSE70947, and GSE86374 together. We found five core genes as common DEGs in the three datasets with significant poor survival. They are *KIF4A*, *RACGAP1*, *HMMR*, *CKS2*, and *SHCBP1*. *KIF4A* (kinesin family member 4A) is a member of the kinesin 4 subfamily. This gene is coding by protein; it is highly expressed in hematopoietic tissues, thymus, fetal liver, spleen, adult thymus, and bone marrow, and lower levels are found in the testis, heart, kidney, colon, and lung [17]. Diseases associated with *KIF4A* include mental retardation, X-linked 100 [18], and retinoblastoma [19]. *HMMR* (hyaluronan-mediated motility receptor) is a protein-coding gene. When hyaluronan binds to *HMMR*, the phosphorylation of PTK2/FAK1 occurs. It may also be involved in cellular transformation regulating extracellular-regulated kinase (ERK) activity and metastasis formation [20]. *HMMR* is expressed in breast tissue and forms a complex with *BRCA1* and *BRCA2*. It is potentially associated with higher risk of breast cancer. Diseases associated with *HMMR* include breast cancer [21] and fibrosarcoma [22]. *RACGAP1* (Rac GTPase-activating protein 1) is a protein-coding gene which encodes a GTPase-activating protein (GAP). It is highly expressed in the thymus, testis, and placenta and lower expressed in the spleen and peripheral blood lymphocytes; the highest levels of its expression were found in spermatocytes while in testis the expression is restricted to germ cells [23]. *CKS2* (CDC28 protein kinase regulatory subunit 2 or cyclin-dependent kinase regulatory subunit) and *SHCBP1* (SHC binding and spindle associated 1 or SHC SH2 domain-binding protein 1) are also protein-coding genes. Gene ontology annotations related to CKS2 and SHCBP1 both include protein binding [24, 25]. The five genes are all coding by protein, so we can detect their protein level in tissues by immunohistochemistry. In our studies, *HMMR* is associated with breast cancer poor prognosis; it is also known for the relationship with *BRCA1* and *BRCA2*. *CKS2*, *KIF4A*, *RACGAP1*, and *SHCBP1* all have positive correlation with *HMMR*. Therefore, they have the possibility to become combination biomarkers as indicator of prognosis for different subtypes of breast cancer especially TNBC and targets of this disease.

However, there were some limitations in our study. First, although high expressions of *KIF4A*, *RACGAP1*, *CKS2*, *SHCBP1*, and *HMMR* were independent prognostic factors of poor OS of breast cancer, all the data in our research came from online database and we need further experiments in vitro and in vivo to validate our findings. Second, there is a lack of external datasets to identify our finding, so false positive may exist. Third, we did not explore the potential roles in diagnosis and therapy these genes played, so more studies are needed and further studies should focus on investigating the underlying mechanism between these core genes and different subclasses in breast cancer especially TNBC.

## 5. Conclusions

The expressions of *KIF4A*, *RACGAP1*, *CKS2*, *SHCBP1*, and *HMMR* were high in breast cancer, associated with poor OS and different breast cancer subclasses. These candidates may provide underlying therapeutic targets to distinguish different subclasses of breast cancer and become clinically diagnostic biomarkers in the near future. *CKS2*, *KIF4A*, *RACGAP1*, and *SHCBP1* all have positive correlation with *HMMR*; they may become combined indicator of prognosis or targets for different subtypes of breast cancer. Our findings reinforce the importance of DEGs in breast cancer and provide new insights into the novel strategies of therapy and prediction of prognosis in different subclasses of breast cancer. It will be useful for further clinical applications in breast cancer diagnosis, prognosis, and targeted therapy.

## Figures and Tables

**Table 1:** Statistic of the three microarray databases derived from the GEO database.

GEO accession	Breast cancer	Normal	Total	Platform
GSE86374	124	35	159	GPL6244 [HuGene-1_0-st] Affymetrix Human Gene 1.0 ST Array [transcript (gene) version]
GSE5364	183	13	196	GPL96 [HG-U133A] Affymetrix Human Genome U133A Array
GSE70947	148	148	296	GPL13607 Agilent-028004 SurePrint G3 Human GE 8x60K Microarray
Total	455	196	651	

**Table 2:** The prognostic information of the 55 hub genes.

Category	Official gene symbol				
Genes with significantly worse survival (*P* < 0.01)	*KIF4A*	*RACGAP1*	*CKS2*	*SHCBP1*	*HMMR*
Genes with significantly worse survival (*P* > 0.05)	*CDK1*	*AURKA*	*CENPE*	*NDC80*	*TACC3*
	*CDC20*	*SPC25*	*BUB1B*	*BUB1*	*CCNB2*
	*CENPF*	*ZWINT*	*DLGAP5*	*KIF20A*	*MAD2L1*
	*TPX2*	*ECT2*	*KIF11*	*NEK2*	*CCNB1*
	*KIF2C*	*KIF23*	*TYMS*	*UBE2C*	*TTK*
	*TOP2A*	*NUSAP1*	*PRC1*	*TK1*	*PBK*
	*CCNE2*	*FOXM1*	*RRM2*	*MCM4*	*SMC4*
	*MKI67*	*ASPM*	*CEP55*	*CENPU*	*MYBL2*
	*EZH2*	*KIF18A*	*RAD51AP1*	*KIF14*	*CDKN3*

## Data Availability

The data used to support the findings of this study are available from the GEO database (https://www.ncbi.nlm.nih. gov/geo/).
